# Blockchains for COVID-19 Contact Tracing and Vaccine Support: A Systematic Review

**DOI:** 10.1109/ACCESS.2021.3063152

**Published:** 2021-03-02

**Authors:** Laura Ricci, Damiano Di Francesco Maesa, Alfredo Favenza, Enrico Ferro

**Affiliations:** 1 Department of Computer ScienceUniversity of Pisa 9310 56126 Pisa Italy; 2 Department of EngineeringUniversity of Cambridge 2152 Cambridge CB3 0FS U.K.; 3 LINKS Foundation 10131 Turin Italy

**Keywords:** Blockchain, distributed ledgers, cryptography, smart contracts, COVID-19, contact tracing, vaccine

## Abstract

Several blockchain projects to help against COVID-19 are emerging at a fast pace, showing the potential of this disruptive technology to mitigate the multi-systemic threats the pandemic is posing on all phases of the emergency management and generate value for the economy and society as a whole. This survey investigates how blockchain technology can be useful in the scope of supporting health actions that can reduce the spread of COVID-19 infections and allow a return to normality. Since the prominent use of blockchains to mitigate COVID-19 consequences are in the area of contact tracing and vaccine/immunity passport support, the survey mainly focuses on these two classes of applications. The aim of the survey is to show that only a proper combination of blockchain technology with advanced cryptographic techniques can guarantee a secure and privacy preserving support to fight COVID-19. In particular, this article first presents these techniques, i.e. zero-knowledge, Diffie Hellman, blind signatures, and proxy re-encryption, then describes how they are used in combination with blockchains to define robust and privacy-preserving solutions. Finally, a brief description of blockchain applications beyond contact tracing and vaccine certification is presented.

## I. Introduction

On the 30th of January 2020, the Coronavirus Disease (COVID-19), an outbreak caused by the virus ‘Severe Acute Respiratory Syndrome Coronavirus 2 (SARS-CoV-2)’, was declared a Public Health Emergency of International concern by the World Health Organization (WHO) [1]. Even if it was referred to as a health crisis first, it has also produced collateral and multi-systemic consequences on healthcare, economic, social, and information systems.

Several areas of society have been affected by the COVID-19 crisis. The economic system is significantly struggling to offset the financial losses caused by the pandemic. This situation will inevitably lead to the closure of many companies and the consequent loss of jobs [2], [3]. The education system is also suffering a severe blow, with abrupt interruption of learning paths of young people, a problem which, according to the United Nations, is involving a large portion of the school-age population in the world.

Even if all the previous areas have been deeply involved in the COVID-19 crisis, there is no doubt that the area that has mainly suffered the fallout of the crisis is that of healthcare infrastructures. The consequences of COVID-19 have particularly caused problems in this area, at different levels. At the supply chain level, with a shortage of medical equipment [4] and evident difficulties of governments and national healthcare services to provide medical staff and population with the minimum medical facilities necessary to face the pandemic and reduce its diffusion. Moreover, the national systems are struggling in performing an accurate prediction of the pandemic course, mainly due to the widespread lack of automation in data sharing between different healthcare structures [5].

Despite the wide applicability of blockchain technology [6], a recently published work [7], highlights that the most prominent uses of blockchains to mitigate COVID-19 consequences are in the area of contact tracing and vaccine/immunity passports support. Also an official document of the European Parliament [8], which recognizes blockchains as one of the ten technologies to fight COVID-19, acknowledges as main current application scenarios infections tracking and health data monitoring. For this reason, we decided to focus this survey mainly on an in depth analysis of these blockchain based applications.

Automatic contact tracing apps have been proposed to detect an individual’s exposure to contagion together with social distancing directives to protect the health of individuals and minimize infections. Most of these apps require the presence of a centralized server, raising serious privacy concerns, as they are susceptible to deanonimyzation and mass surveillance attacks.

The recent availability of vaccines for COVID-19 makes it urgently necessary to consider proper infrastructures for vaccine delivery and deployment of vaccination e-certificates. Indeed, even if, during the first phases of the pandemics, WHO [9] did not recommend the deployment of “immunity passports”, because there was no evidence of a permanent immunity given by the infection of COVID-19, in December 2020, with a view on several coming COVID-19 vaccines, WHO [10] is suggesting to use e-vaccination certificates.

Both contact tracing apps and vaccination e-certificates have posed doubts on the protection of some of the fundamental rights of citizens and the possible “Big brother” effect generated by the contact tracing traces and vaccinations recording by the authorities [11]. This makes it urgently necessary to find proper technologies able to improve the level of security and privacy for these applications.

The aim of this survey is to investigate how blockchains, and, more in general, Distributed Ledger Technology (DLT) can be adopted in the scope of social and health measures aimed at reducing the spread of the COVID-19 infection to allow a return to “normality”. To this aim, this study mainly focuses on a deep analysis of several blockchain-based approaches for contact tracing and for immune/vaccine certifications, analysing their strengths and weaknesses and how they can perform as effective tools to monitor and combat the spread and impact of the disease.

Even if some surveys on the use of blockchain for mitigating COVID-19 consequences have been recently presented [12]–[16], all of them present a general, high level description of the architectures of the blockchain-based systems. Instead, our aim is to present an in depth analysis of how blockchain technology can be enhanced with advanced cryptographic tools to guarantee secure and privacy preserving supports for fighting COVID-19, with the goal of defining applications respecting the fundamental rights of citizens. In particular, we focus on the automation of contact tracing and e-certificates management.

This article is organized as follows. Sect. II introduces the background on blockchain technology and cryptographic techniques. The solutions for blockchain-based contact tracing are presented in Sect. III, while those for immune/vaccine certifications in Sect. IV. Other blockchain-based proposals to face COVID-19 consequences are briefly presented in Sect. V. Sect. VI contains a discussion of the open problems and Sect. VII presents related works. Finally, Sect. VIII draws the conclusions.

## II. Background

In this section we introduce the essential background to understand the blockchain-based solutions presented in the following sections. Sect. II-A introduces the basic concepts of blockchain technology, while Sect. II-B presents the cryptographic protocols used in the considered blockchain-based proposals.

### A. Blockchains and Smart Contracts

Blockchains and, more in general, distributed ledgers, are a new disruptive technology introduced in the last decade. They allow the management of a tamper free ledger shared between several entities in an untrusted environment. The ledger can store a collection of records, like cryptocurrency transactions in Bitcoin [17], the events occurring in a supply chain or the state of a set of smart contract, like in Ethereum [18]. The ledger can be stored in a chain of blocks, or in more complex data structure, like a directed acyclic graph (DAG) (first proposed in [19]), where the tamper freeness of the ledger is guaranteed by a cryptographic protocol. In the following, we will refer to distributed ledgers stored in chains of block, i.e. blockchains, because this is the structure exploited by all the solutions we will present.

To decide which blocks have to be added to the blockchain, a distributed consensus algorithm is executed which guarantees that, under certain conditions (often related to the percentage of honest participants), a consistent and correct version of the blockchain is updated and shared by all the participants. The tamper freenes guarantees that the blocks of the blockchain cannot be changed, providing persistency (information remains publicly visible), timestamping (information exists at a given discrete time), and immutability (information can not be changed). These properties altogether provide auditability, i.e. it is possible to prove that a given information does exist at a given time and is not changed later.

An important breakthrough in blockchain technology has been achieved with Ethereum [20], a blockchain platform able to execute smart contracts, i.e. stateful applications executed by all the nodes participating to the peer-to-peer network, without involving third parties. Smart contract are written in a Turing-complete programming language, which may be domain specific, like Solidity, or general purpose, like C++, and executed on the Ethereum Virtual Machine (EVM). The execution of a smart contract updates the state of the blockchain only if the majority of the nodes agrees on it through the consensus algorithm.

Several types of blockchain have been proposed in the last years, which may be classified as permissionless/permissioned and public/private. The first dimension distinguishes between blockchain whose governance, which is mainly related to the set of nodes allowed to participate to the consensus, is opened to everyone from those which restrict it to a set of authenticated users. The second dimension, public versus private blockchains, regards the choice to enable any node to read the information stored in the blockchain or to restrict it. Public blockchains are good in terms of transparency but may not suit, for instance, the needs of a company, that obviously cannot allow unknown entities to view the transactions of its customers. Instead, a public administrative office may adopt a permissioned public blockchain to keep the control of the registration of transactions on the blockchain, while making all the transactions public and accessible to all citizens, to provide transparency and auditability.

### B. Cryptographic Techniques

This section introduces the basic cryptographic tools used by the proposals described in the next sections.

**FIGURE 1.**
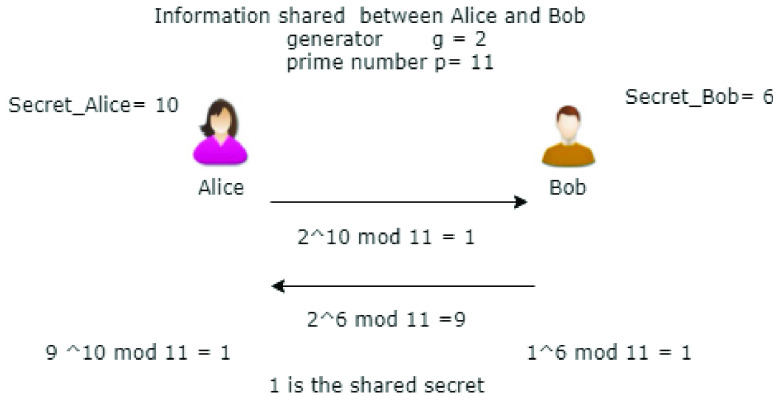
The Diffie Hellman protocol at a glance.

The *Diffie Hellman protocol (DH)* [21] is generally used when two entities, connected by an insecure channel, want to share a secret key, which may be needed, for example, to encrypt a message with a symmetric encryption algorithm. The two entities share two numbers, a prime number $p$ and a generator $g$ of the group $Z_{p}$. Figure 1 shows a simplified version of the DH protocol: each entity first generates a secret $s$ and then exchanges with the other entity a value computed from $g$, $p$ and $s$. After the exchange, the two entities are able to compute a secret key only known to them. The protocol is secure if and only if the Decisional Diffie–Hellman (DDH) assumption holds, i.e. the assumption which guarantees computational hardness of discrete logarithms in cyclic groups.

A *zero-knowledge proof* is a cryptographic mechanism [22] by which one entity, the prover, can show to another party, the verifier, that they know some information (e.g. a simple value or the correct execution of a program on a set of inputs), by only proving the knowledge of it, without revealing any additional information on the information itself. The first proposals of zero-knowledge protocols envisaged multiple rounds of interaction back and forth between the prover and verifier. In the last years, the diffusion of blockchain has offered an incentive for the definition of more scalable and efficient protocols, like zk-SNARKS, i.e. “Zero-Knowledge Succinct Non-Interactive Argument of Knowledge” [23]. The acronym refers to the fact that the protocol requires a single round of interaction between the prover and verifier and that the length of the proofs and the complexity of their execution are reduced so to make it possible to integrate these techniques in the blockchain. Several implementations of zk-SNARKS currently exist [24], [25] and can be integrated with blockchains (e.g. [26]).

*Blind signatures*, introduced by Chaum [27], are a kind of digital signature where the content is disguised before it is signed by a third party unable to inspect the content. After that, the content may be revealed, and the signature appear on it as a normal digital signature. Blind signatures are generally employed in privacy critical protocols, where the signer and content generator are different parties, and the privacy of the content is important. Electronic-election systems and digital cash schemes have been among the main applications that have seen their adoption.

**FIGURE 2.**
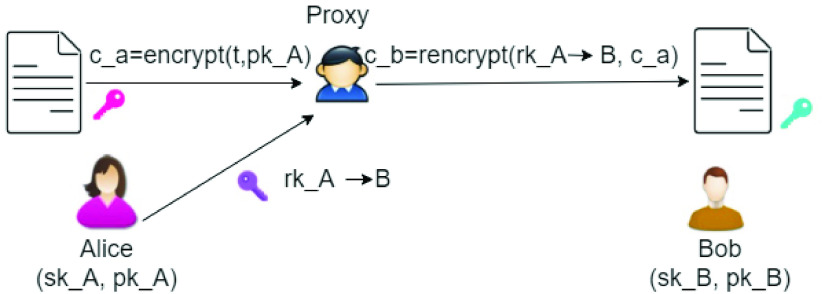
Proxy re-encryption.

Finally, *Proxy re-encryption* [28] is a type of Public Key Encryption technique that allows a proxy to re-encrypt data encrypted with one public key $K_{1}$ to another public key $K_{2}$, without having access to the underlying plaintext or to the private key corresponding to $K_{1}$. Consider the example in Figure 2: Alice has encrypted a document $t$ with her public key $pk\_{}A$, and has sent the encrypted document $c\_{}a$ to a Proxy, which may be a cloud provider or an IPFS [29] node. Afterwards, Alice decides to delegate the access to the document to Bob, who owns a pair of asymmetric keys $sk\_{}B, pk\_{}B$. Instead of decrypting the document with her private key and re-encrypting it with Bob’s public key, Alice creates a re-encryption key using her secret key and the public key of Bob and sends it to the Proxy. The Proxy will re-encrypt $c\_{}a$ by using the re-encryption key, so obtaining a new encrypted document $c\_{}b$. Bob can then decrypt $c\_{}b$ using his secret key.

## III. Blockchain for Contact Tracing

Before presenting how blockchain technology can support and enhance contact tracing, we briefly summarize the main approaches for tracing contacts recently proposed to cope with the COVID-19 outbreak.

Contact tracing is the process of identifying individuals that may have been in contact with an infected persons to notify them the possibility of infection. The idea of using contact tracing for tackling epidemics dates back to the fourteen century, when the idea of quarantine was introduced to reduce the black plague infection [30]. In more recent times, manual contact tracing has been used by interviewing infected individuals to detect the people they have recently been in contact with. Manual contact tracing presents the evident drawbacks of being slow and requiring relevant manpower. This can be avoided if the same goal is achieved by taking advantage of the rich set of mobile communication technologies currently available.

Below we briefly list the main technologies currently exploited by contact tracing apps, while we refer to [31] for a more in-depth analysis.

• **Proximity-based Contact-tracing (PCT).** The proposals falling in this area are based on detecting the *relative positions* of smartphones. Most of them employ the BLE (Bluetooth Low Energy) technology and exploit the Blue-Trace protocol [32]. Many contact tracing apps are currently based on this solution, an in-depth analysis of these apps is presented in [33].
  Even if BLE is intrinsically a distributed protocol, which enables peer-to-peer interactions between the mobile nodes, many solutions, like TraceTogether (Singapore) [34], CovidSafe (Australia) [35], and the solutions based on the PEPP-PT model [36], like StopCovid (France) [37], use a centralized server, so introducing privacy threats.
  The most serious privacy problems characterize the solutions where the central server generates a Temporary ID (TID), comprising the UserID, the creation and the expiry time, for each device registered to the service, and then encrypts the TID symmetrically with a secret key which is known only to the central health authority. The TIDs are then exchanged between the mobile phones, to register their encounters. The health authority uploads to the server the TID of an infected user together with the TID of the other users they have encountered. Even if the possibility of replay attacks is minimized by reducing the validity of each TID to 15 minutes, this solution raises several privacy concerns. Indeed, the server is able to decrypt the identities of all individuals at risk, to send them a warning.
  On the other side, the “Decentralized Privacy-Preserving Proximity Tracing” project, DP3T, [38] proposes a decentralized approach which enable mobile phones to autonomously generate a set of pseudonyms, which are exchanged between phones in close proximity, without the intervention of a central server. However, even these applications exploit a centralized server, which, in this case, only acts as a “rendez-vous” point where infected users upload their pseudonyms, while other users download them from the server and autonomously find potential matching with infected users. As we will discuss in the following, the function of the server may be carried out by a blockchain, so enhancing the transparency of the whole process.
• **Location-based Contact Tracing (LCT).** In this class of solutions, contacts are detected by exploiting the *absolute locations* of the smartphones, returned by the GPS or by WiFi access-points. Only a few countries, like Iceland and India, are currently employing LCT apps. A drawback of these approach is that current location mechanisms, like GPS, are not secure, because nodes could easily provide fake information. Furthermore, this solution may present serious privacy problems.
• **Mobile Operator Contact Tracing (MOCT).** Mobile operation location tracking exploits the mobile operator’s infrastructures, like base stations of cellular networks, to locate cell phones. This is the solution adopted in Israel, which has tracked all citizens during the COVID-19 pandemic. Its main drawbacks are low accuracy and the privacy risks. For these reasons, it is not generally used to perform contact tracing, but rather to evaluate the impact of the lockdown measures and to detect potentially contagion hotspots.

A more comprehensive discussions of the advantages and drawbacks of the previous solutions is presented in [31].

In the following sections, we discuss how blockchains can improve the effectiveness of each one of these technologies.

### A. Blockchain Support for PCT Solutions

The proposals described in this section enhance proximity tracing through the blockchain technology. We only consider decentralized solutions, since the centralized ones are strongly based on the trust of users in a central authority, which is just the opposite approach of the one behind blockchains.

The main feature of the following proposals is that they exploit BLE to exchange pseudonyms of the mobile phones coming into close contact and exploit the blockchain as a bulletin board for notifying new infections. The solutions are characterized by different level of privacy and differ in the techniques used for generating the users’ pseudonyms.

**FIGURE 3.**
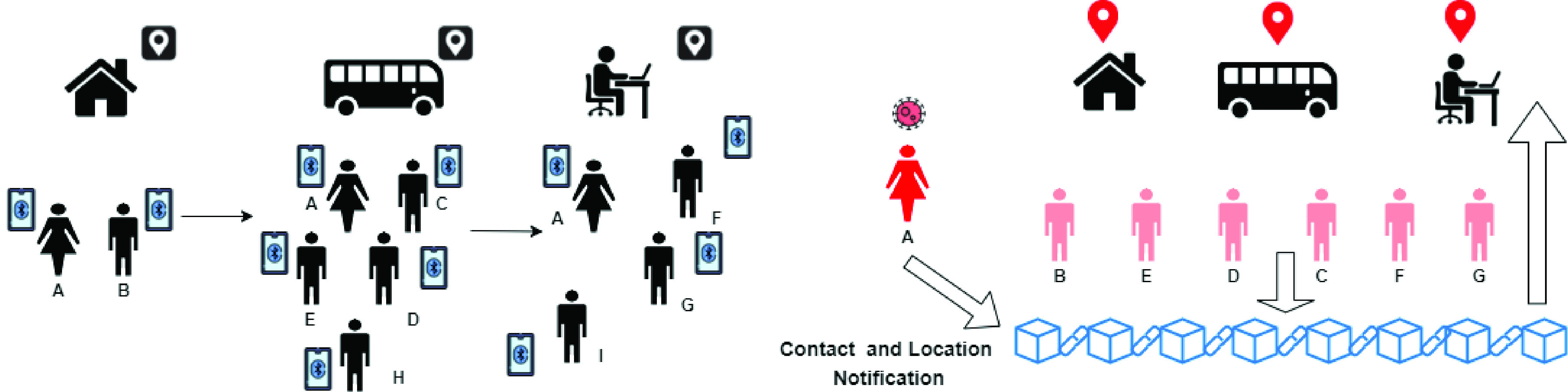
Contact and location tracing.

Authors of [39] present a system unifying, in a single blockchain-based framework, PCT and LCT. The individual tracing system focus on person-to-person contact via BLT. Since WHO declared that the virus could survive on material surfaces [40], the authors propose also a location-based tracing system supported by a set of smart contracts. The proposal is schematically shown in Figure 3. The left part of the Figure shows a scenario where the user A visits several locations, i.e. home, bus and office, afterwards is detected infected. As shown in the right part of Figure 3, the support enables each person who came in close contact with A to detect the possibility of contagion (note that H and I are not at risk because their distance from A has not been considered at risk) and the location visited by A are tagged as infected, while the users at risk are depicted in pink. Both actions are supported by a blockchain, ad described in the following.

In this section, we describe the system component of [39] which manages PCT, while the LCT part of the system will be described in the next section.

The mobile phones coming in contact exchange randomized mac addresses by BLE. Contact information includes, besides the close phone’s mac address, the start and end time of the interaction and the strength of the received signal, and may be recorded on the mobile phone or on the blockchain. When a user U becomes infected, they broadcast a transaction containing their health status update alongside all the BLE randomized mac addresses they have generated in the past 14 days (older addresses are no more useful, because the incubation period of COVID-19 is at most 14 days). Other users can check the local copy of the blockchain and verify if they have been in contact with U.

To guarantee privacy, authors suggest to use Bluetooth Random Private mac addresses, that are randomly generated and frequently modified by the Bluetooth protocol. A higher level of privacy is guaranteed by increasing the number of identifiers, which, on the other hand, increases also the network traffic and the blockchain load. The challenge is to define the number of identifiers exchanged by the mobile phones to obtain a proper balance between the two factors. A similar approach is proposed in [41].

The main advantage of this solution is that it avoids the use of a centralized server which may tamper with the pseudonyms uploaded by the infected citizens, by using instead a blockchain. However this solution fails to solve many privacy attacks which affect BLE-based solutions [42], [43]. Indeed, despite the use of dynamic randomized mac addresses helps to increase the anonymity of users, it is still vulnerable to several privacy threats. Consider, for instance, the Paparazzi attack [33] whose goal is to deanonymize an infected user U. The attacker installs a set of passive BLE devices, i.e. devices only able to receive BLE signals, in strategic positions, for instance along the way U uses to go from home to work. When the user U, target of the attack, is detected positive, they upload all their randomized mac addresses on the blockchain, and the attacker may compare the addresses it has collected with those uploaded on the blockchain and so deanonymize U. As shown in [44], more sophisticated attacks may be organized to implement a real mass surveillance strategy.

Another strong assumption made in [39] is that users always honestly upload their infection status on the blockchain. This is not a realistic assumption, since malicious users could upload fake status updates on the blockchain with the goal of provoking panic in the population. Finally, the possibility of dynamic updating the mac addresses is currently not fully supported by current operating systems for mobile phones.

A more robust proximity-based contact tracing proposal is PRONTO-C2 [44], an interesting proposal combining the Diffie-Hellman (DH) secret sharing protocol with a bulletin-board implemented through a blockchain. A similar approach is also proposed in [45]. The protocol can be described through a simple metaphor: PRONTO-C2 enables users to autonomously and confidentially call each other to alert the presence of a detected infection (note that the Italian word “Pronto” stays for “Hello” and C2 pronounced in English stays for “is you” in Neapolitan language). This is obtained by properly applying the DH protocol. To this end, the pseudonym of each user is a group element in a setting where the Decision DH assumption holds. The basic idea of the protocol is to replace the generation of users’ pseudonyms with that of *unique encounter identifiers* generated by applying the DH protocol. An encounter identifier is a secret key $K$, which is computed by applying DH, and is shared only by the two mobile phones which have been in close contact. The protocol defines a mechanism enabling the users detected as infected by the health authority “to call” all the contacts with whom they have shared the secret in a secure and privacy-preserving way. After having received an authorization from the health authority, they upload the secret keys, which uniquely identify each of their encounters, on a blockchain acting as a bulletin-board. Users can periodically check the blockchain to verify if some of the keys in their possession have been published on it.

**FIGURE 4.**
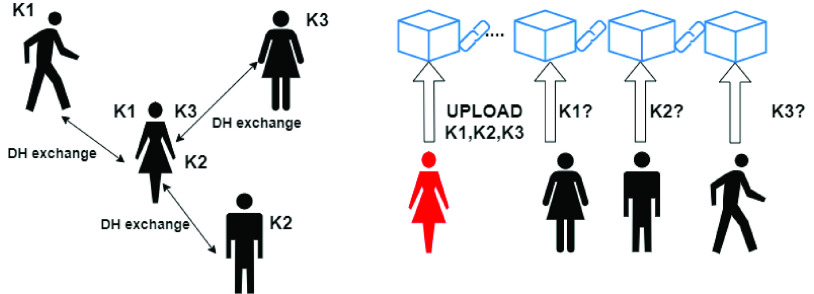
The ProntoC2 protocol.

Figure 4 presents an outline of the protocol: in the left part, an individual comes into contact with three other people and shares different secret keys, respectively K1, K2, and K3, with each of them. The right part of the Figure shows that, when that individual is detected infected, all their encounter keys are uploaded on the blockchain. The other ones query the blockchain to check if their own secret has been published.

Note that an attacker can only intercept the single messages of the DH protocol, but cannot steal the secret keys, which are known only to the users that came in contact with each other, so the information published on the blockchain is not linked ad, thus, can not help deanonymization attack attempts.

Of course, to prevent Denial of Service (DoS) attacks (or panic spreading attempts), only users authorized from the health authority can upload their identifier on the blockchain. To prevent the government from linking patients to information on the server, PRONTO-C2 suggests to use *blind signatures*. The health authority releases an authorization code to the infected users, which are sent to the laboratory from the government. The infected user then exchange the authorization code with a blind signature which can be verified on the blockchain through a smart contract.

Note that the implementation of the bulletin-board with a public blockchain guarantees the transparency of the whole process. Indeed, the encounter identifiers are meaningful only for the users involved in the contacts with the infected one and the blind signature guarantees the anonymity (inside the tested population) of the identities of the infected users.

The main problem of the proposal is that the DH protocol requires elements of at least 256 bits for the group element and this may exceed the size of the Bluetooth identifier beacon. For this reason, the authors have recently proposed a lighter version of the protocol, PRONTO-B2 that does not require to translate the beacon identifier in to a group element.

A solution for contact-tracing, similar to the first one presented in this section, as far as concerns the advertisement on the blockchain of the infected users, is that of [46] which is affected by similar privacy problems. The extra contribution of [46] regards the use of the blockchain to enhance the control of the pandemics. The author observes that current proximity-based contact tracing solutions do not enable a global view of the outbreak evolution, which may be useful, for the government and for the citizens. Indeed, all the relevant information about encounters is stored on the users’ mobile phones, making it unfeasible to obtain aggregated information. As a first solution, [46] suggests to exploit a public blockchain where each user can upload synthetic information about each of their *qualified encounters*, i.e. encounters with another phone within a certain distance and lasting a certain period of time. While a personal record of the encounter includes the pseudonymous of the phones and the time and duration of the contact, and is used to perform contact tracing, a *redacted record* reports information to compute aggregated statistics. A redacted record may contain, for instance, only the information that a mobile phone has had at least one qualified encounter. The list of the redacted encounters is stored on a public site, while its hash is published on a blockchain so that all the citizens can access and check the integrity of the information.

The list of redacted encounters may be simply replaced by the number of qualified encounters of a mobile phone. Note that also a minimal amount of aggregated information, for instance only the number of encounters, may be very useful for the government to guide the governance of the outbreak. For instance, it is possible to follow the trend of the contacts increase, due to the re-opening of some activities, like, for instance, discos and other entertainment venues. As far as concerns the scalability of the proposal, [46] suggests to ease congestion of the blockchain by posting on it only the hash of the encounters list. Furthermore, scalability is also guaranteed by the *cryptographic sortition* technique of Algorand [47].

A working web-app, iReport-Covid, has been developed [48] to share COVID-19 data on the Algorand blockchain [47]. The app suggests the users to compile a survey about their experience, if they have been infected. Data provided by the users are registered on the blockchain, where they can never be removed or changed, so they are shared in a transparent way.

### B. Blockchain Support for LCT Solutions

LCT solutions use absolute geographic locations of mobile phones to perform contact tracing. One of the main problems of these approaches is maintaining *user’s privacy*, because location data can be easily used to violate the private lives of citizens. Another problem is *location forging* that can be easily achieved by exploiting, for instance, the GPS open APIs available for smart phone operating systems.

The proposal [39], which has already been considered in the previous section as for the contact tracing system, defines also a location-based tracing based on a hierarchy of smart contracts, paired with hierarchical administrative domains, e.g. state, region, city. The smart contracts are invoked by the users, when they check in an area, to control if that area has been infected. Furthermore the users voluntarily notify their health status to the smart contracts relative to the areas visited in the last 14 days. The smart contract automatically reverts the status of the location to non-infected as soon as the contagion period is expired. An incentive mechanism is defined to encourage individuals to use the system. This may lead to several attacks, as will discuss in Sect. VI.

The *BeepTrace* proposal [49] is mainly focused on defining mechanisms to guarantee user’s privacy in the whole contact tracing cycle. Several positioning technologies (GPS, Bluetooth, Cellular network and WiFi) are used together with two blockchains, a *tracing* and a *notification* blockchain. The tracing system as a whole is based on the collaboration of several parties, users, diagnosticians, Certification Authorities, geodata solvers and positioning service providers which interact through both blockchains.

**FIGURE 5.**
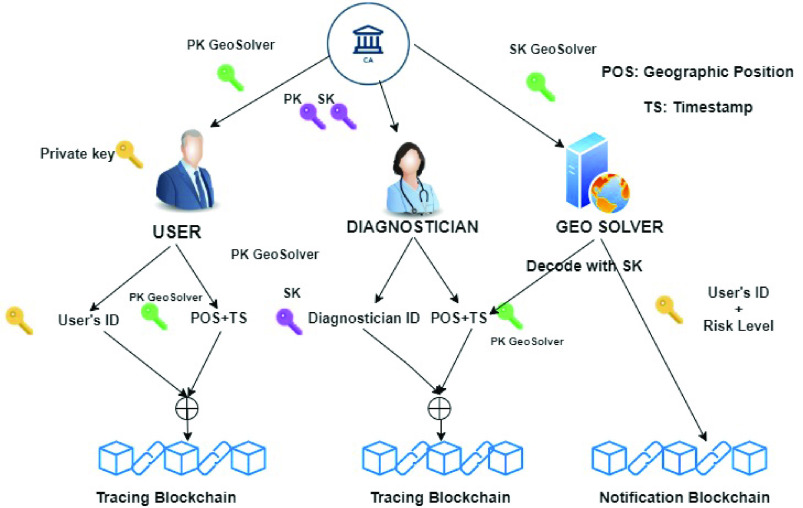
The BeepTrace protocol.

We refer to Figure 5 to show how contact tracing is implemented in *BeepTrace*:

• at bootstrap, a certification authority distributes asymmetric key pairs to the authorized diagnosticians and to the geodata solver authority. The geodata public key is also distributed to the users.
• the app installed on the users’ phones periodically generates a *TraceCode*, obtained by encrypting their identifier with a private key and concatenating the identifier with their location and timestamp information. The resulting TraceCode is uploaded on the Tracing Blockchain. A local private key, which is refreshed daily, is used to generate the user’ pseudonym, while the location and timestamp is encrypted using the public key of the geodata solver authority.
• when the trusted health authority diagnoses an infected user, it collects from them their recent TraceCodes and verifies, through the user’s private key, the real ownership of the pseudonyms. The user identity is revealed to the diagnostician, but this step is protected from regulations and laws, like the GDPR. On the other hand, this guarantees that information about infected users is shared responsibly, and prevents the diffusion of fake pseudonyms which may produce panic in the population. To further enhance users’ privacy, the trusted health authority replaces the pseudonym in TraceCode, with their pseudonym, signed by their private key.
• at this point, the geodata solver decrypts the location information from the TraceCodes certified by the health autority and stored in the tracing blockchain, and performs location matching. The geodata solver uploads pseudonyms of the users at risk on the notification blockchain, together with a risk level. Users can access the notification blockchain to check the presence of their pseudonyms and, if present, of the risk level.

A problem to be faced in Location Based Tracing is that of location forging. The blockchain can be exploited to face this phenomenon, by providing *Proof-of-location* (PoL) mechanisms, i.e. certifications of the users’ presence at a location at a certain time. Reference [50] originally proposed Proof-of-Location based on blockchains, for location-based-services, like location-based rewards, recommendations or social networks gaming. Short range-communications are exploited to enable *provers*, i.e. nodes that need a certification of their location, to collect proofs of location from their neighbours, called *witnesses*. The proofs of location are stored in a blockchain whose consensus algorithms is a modified version of Proof of Stake, which favours the election of nodes according to the number of PoL they have registered in the latest T blocks of the blockchain.

**FIGURE 6.**
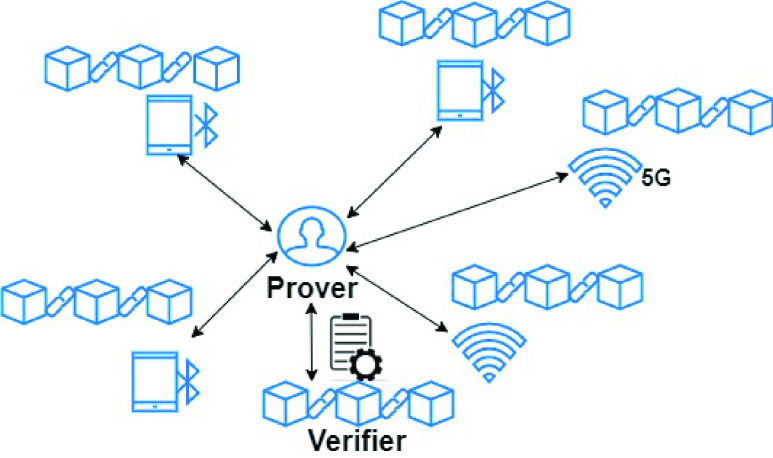
Blockchain for proof of location.

More recently, PoL has been proposed for contact tracing. In [51], Bychain, a permissionless blokchain for location based tracing is presented. Witnesses may be WiFi Access Points, nodes equipped with BLE, or LTE base stations owned by an Internet Service Provider, all equipped with GPSs and identified by a couple of public-private keys. The node which needs a certification (prover) collects a set of proves from close witnesses, combines them, and registers them on a blockchain, together with a trust level given by the number of proves received. A smart contract may certify the trustfulness of a Proof of Location, without breaking the user’s privacy, by exploiting an interactive zero-knowledge protocol. Figure 6 shows an overview of the system. As we will discuss in Sect. VI, it is realistic to suppose that the witnesses share their resources (e.g. bandwidth) for PoL services only if a proper incentive mechanism is provided, for instance by implementing a token-based rewarding system on the blockchain.

### C. Blockchain Support for MOCT Solutions

[52] proposes *PriLok*, an infrastructure that should be managed by a state in collaboration with other entities, like telecommunication companies, public administrations and health authorities. The basic idea is to use the cellular network to promote inclusion, since a part of the population, generally aged people, may not own a smartphone or is not able to use Bluetooth. This is even more true in less developed countries. Furthermore, cellular networks are considered more reliable with respect to GPS or Bluetooth.

*PriLok* is defined as an overlay laying on existing infrastructures which adds several functionalities to them. Contact tracing is performed by registering a *Proximity Detail Record* detailing, for each region, the continuous period of time spent by a phone in that region. The *PrilLok* Data Vault is the main data repository which is distributed among several authorities. PriLok requires that a quorum of independent entities reach consensus for all critical operations. Both classical solutions, such as Byzantine fault tolerant protocols (e.g., PBFT [53], MinBFT [54], CheapBFT [55], and modern blockchains [47], [56], [57] can be exploited.

### D. Contact Tracing Proposals: a Comparison

A summary of the main proposals dicussed in the previous sections is presented in Table 1. We can notice that most proposals use the blockchain as a bulletin board. Most of the proposals exploit Bluetooh, while only a few of them rely on smart contracts and, in such cases, the Ethereum blockchain is generally used, with Solidity as the language chosen for smart contracts development.

**TABLE 1:** Blockchain-Based Contact Tracing Solutions

Ref.	Communication Infrastructure	Cryptographic Techniques	Involved Parties	Blockchain Type	Blockchain Role	Smart contracts
[39]	Bluetooth, GPS	Randomized Bluetooth	Mobile Service Layer	Permissionless	Bulletin Board, Location Tracing	✓
[46]	Bluetooth	Chriptographic Sortition	Government	Permissionless	Aggregated Data Board	–
[44]	Bluetooth	Diffie Hellman, Blind Signatures	Health Authority	Permissioned	Bulletin Board	–
[49]	GPS, Bluetooth, Wifi, Cellular Network	Symmetric/Asymmetric Encryption	Health Authority, Certification Authority, Geo-data Solver	Permissioned	User Tracing, Notification	–
[51]	Bluetooth, GPS, LTE, WiFi	Zero-knowledge	Fully Distributed	Permissionless	Bulletin Board	✓
[52]	Cellular Network	–	National Structure involving Telco operators	Permissioned	Data Vault	–

## IV. Blockchain Support for Immune and Vaccine Certification

In this section we will discuss how blockchain can support the management of immunity passports and vaccination certificates which are official documents certifying different aspect of the users’ health. An immunity passport is an official document certifying that an individual has been infected and then recovered from COVID-19, and they have likely developed antibodies for SARS-CoV-2. Anyway, it is much safer for the immune system to learn how to protect you from diseases through vaccination than by catching the disease and attempting to treat it. Furthermore, vaccination gives a strong proof of immunity, as shown by several vaccines recently produced, whose first trials suggest they are highly effective for preventing the infection.

As far as concerns immunity certificates, the topic of their effectiveness is currently being debated [9]. Even if in April 2020 WHO affirmed that there is not enough evidence about the immunity generated by the COVID-19 infection, some countries, like Estonia, developed a digital “immunity passport” app [58] allowing users with antibodies to show their reduced risk of spreading the virus. In early April 2020, the health secretary of the United Kingdom introduced, for the first time, the idea of *Immunity Passport* as a mean to enable people to come back to work [59]. A strictly related theme is that of public transport, as one of the main contexts where the contagion may spread. Reference [60] proposes to emit Antibody certificates to allow immune citizens to travel on public transports, with the goal of returning to work. Reference [60] investigates also another interesting use of antibody certificates, that is to use them to reduce the risks related to food/goods delivery services for aged/vulnerable people. In this case, requiring an antibody certificate to the good carrier may reduce the risk of infection for these people.

An initiative of the Greek Government [61] proposes *Digital Health Passports* [62] certifying risk-free individuals, i.e. individuals not actively carrying the virus. The idea originates from the initiative which required a certification for travelers entering Greece, attesting they have been tested to be COVID-19 free at most 72 hours before their departure. This initiative may be framed in the context of measures trying to contain the spreading of the pandemic, while reducing its negative impact on the economy, that, in Greece, is mainly based on tourism.

In all the cases, an efficient structure enabling fast access and a simple management of certifications is urgently required to safely admit individuals in social activities and travels.

In the next sections we first introduce the idea of Distributed Public Key Infrastructures and of Verifiable Credentials as two building blocks, based on blockchain technology, for the definition of a certification system. We then present a set of recent proposals targeted to the COVID-19 scenario.

### A. Decentralized Verifiable Credentials

The problem of defining a standard for digital certifications and credential predates the COVID-19 outbreak. A *verifiable credential* or *verifiable claim* is a piece of information that a third party can validate digitally, in a secure and privacy preserving way. Verifiable credentials support *self-sovereign identity*, that means that the identity owners accumulate credentials into an identity account and use the credentials to prove some property to verifiers, revealing the minimal amount of information necessary for the verification.

The “W3C Verifiable Claims Working Group” of the WWW Consortium, presented, in November 2019, a standard called “Verifiable Credentials Data Model” [63]. They define a standard document format for certification, and, more important, propose a new distributed architecture for Public Key Infrastructures, responsible for the authentication and the distribution of public keys, which may greatly benefit from blockchain technology. The idea is to use the blockchain as a register to store the correspondence between the *Decentralized Identifiers (DIDs)* and their public key. The control of a DID is managed through the DID’s private key. So doing, the blockchain takes the role of the registers managed by the centralized Certification Authorities. Note that DID may represent individuals, but also communities, states, companies, connected objects, etc. The use of blockchain may help to solve many of the problems of centralized PKI. For instance *identity retention*, i.e. preventing a user from registering a public key under an identity which is already been register, is not always ensured by current centralized PKIs, while it may be guaranteed by the blockchain consensus protocol [64]. Several blockchain-based platforms supporting the verifiable credential data model are currently available, like Sovrin [65], which is based on Hyperledger Indy and uPort [66], which exploits Ethereum.

**FIGURE 7.**
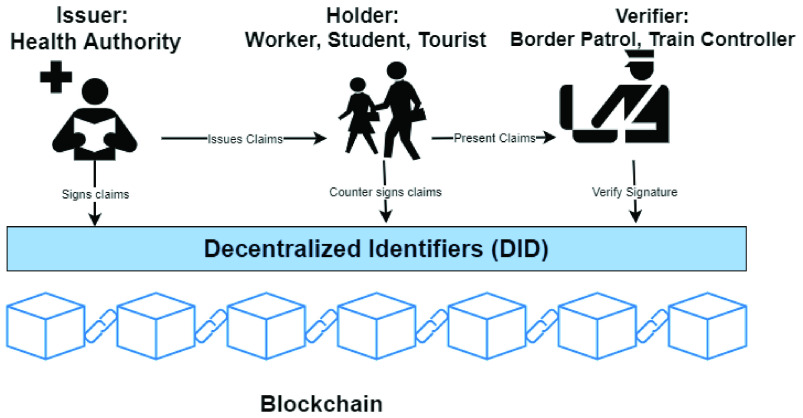
Distributed identifiers and verifible claims.

Several credentials may be assigned by different issuers to the entities whose identity is registered on the blockchain. For instance, the issuer of a COVID-19 certificate may be a public health office, which distributes antibody credentials to immune citizens. The citizen presents their credential to the interested parties, i.e. public travel authorities, airport authorities and so on, which verify them. An example is shown in Figure 7. Note that the certificates assigned to a user are not necessarily stored on blockchain. To preserve user’s privacy, the credentials may be stored in a personal wallet or in a personal cloud storage or also, encrypted, in a distributed file system like the InterPlanetary File System (IPFS) [29], [67]. The blockchain is used by the verifier to find out the public key of the issuer which enables the verification of the claim, through its signature. Furthermore, the issuer may store the *hash* of the document on the blockchain, to enable the verifier to check the *integrity* of the data.

### B. Blockchain-Based Proposals for COVID-19 Certifications

The idea of using verifiable credentials on a distributed infrastructure for defining a immunity/vaccine certification system is exploited by [68]. The verifiable credential is, in this case, the claim that the individual has been vaccinated. When a vaccination or a blood test for immunity is performed, the issuer, which is, in this case, a representative of the National Health Service, first authenticates the holder, then provides a Verifiable Credential which is digitally signed by both the issuer and the holder. The Verifiable Credential is stored on a Consortium blockchain based on a Proof of Authority consensus mechanism [69]. The holder can now present a provably valid certificate to the verifier, which may be the airport or school authority, and so on. This proposal exploits the openEthereum platform, which is a Consortium blockchain. The system exploits zero-knowledge proofs to minimize the information which is sent to the verifiers. The authors also present an app, which is used to generate DID for the certificate issuer and holder.

Authors of [70] present a system which combines decentralized identities, smart contracts and IPFS as off-chain storage for documents, to manage COVID-19 certifications in a decentralized way. The actors of the system, i.e. the Ministry of Foreign Affairs and of Public Health, the COVID-19 testing centers, and the citizens, use the system to manage the *digital health passports*, which record information on citizens’ travel history, immunization, vaccination records, and so on. The Ministries of Foreign Affairs and of Health are the entities authorized to give verifiable credentials to testing centers and health authorities or to revoke them.

Each entity is associated with a smart contract on the Ethereum blockchain. Furthermore, a smart contract is paired with every citizen and includes only the hash of their certificate, while the certificate encrypted with their public key is stored in IPFS. Since the citizen will have to provide proof of vaccination by showing the certificate to different authorities, they delegate the Proxy, which in this case is the IPFS node, through a *proxy re-encription* scheme scheme. As discussed in Sect. II, proxy re-encryption is a type of public-key encryption which enables to transform a text encrypted with a given public key to a text encrypted with another public key, without requiring the knowledge of the hidden plain text. This mechanism, integrated with a blockchain supporting a decentralized key management infrastructure, enables users to encrypt and store their private documents on IPFS and to grant access to authorized users, without the need of new encryption of the data for each new authorization.

**FIGURE 8.**
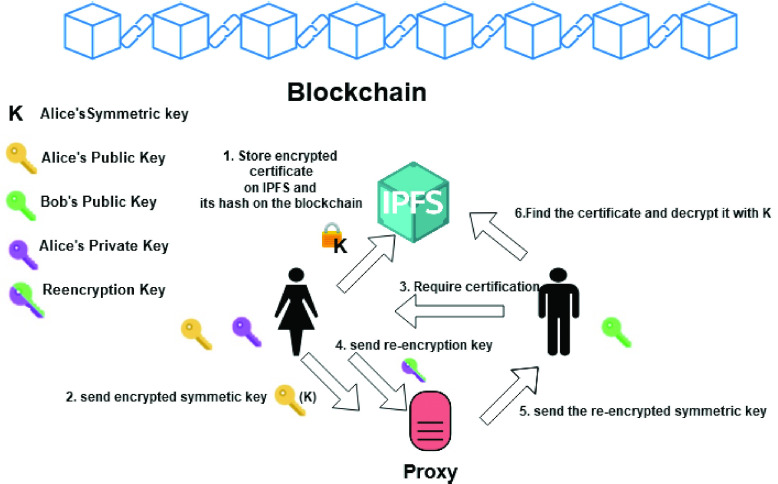
The architecture of the system presented in [70].

Figure 8 shows the operation flow of the system which exploits both symmetric and public key cryptography. Alice recives a certification, encrypts the document with her symmetric key and stores the result in IPFS (1). Furthermore, she sends the symmetric key encrypted with her public key to the proxy (the IPFS node) (2). Suppose Bob is a border control authority which checks Alice’s certification to admit her in the country. Bob sends a request to Alice (3), which retrieves Bob’s public key from his DID, which is stored on a blockchain (for instance Sovrin exploits a blockchain to store DIDs). Then, Alice computes the re-encryption key (from her private key and Bob’s public key) and sends it to the proxy (4), which uses this key to re-encrypt the symmetric key previously ciphered by Alice, without accessing the secret key (5). Finally, the re-encrypted key is used by Bob to decrypt Alice’s symmetric key which enables him to accesses IPFS and retrieve and decode the document (6) and check its integrity through the blockchain.

Authors of [71] propose an online “ COVID-19 Passport” reporting the vaccination status of a citizen. A special feature of this work is that each user is uniquely identified by considering information that the “user knows” (like gender and Date of Birth) and biometric information that the “user possesses” (like users’s iris scans). When a user presents themselves to the health organization, a unique blockchain identifier is generated by considering both information and a record with the vaccination history is stored or updated on the blockchain. Using the biometric information is a good idea, but requires to address the problem that different scans of the same individual may be slightly different. For this reason, techniques like SHA-256 or SHA-3 cannot be used, because, due the properties of the chryptographic hash functions, even a small difference in the input data of the function returns a completely different hash value. Reference [71] suggests to address this problem by using a Locality Sensing Hashing, LSH, technique [72], able to generate similar hash for similar input data, i.e. putting all the similar biometric scans in the same bucket which may be paired with a single user.

Reference [73] proposes to use a blockchain managed by government to store COVID-19 antibody certifications. The blockchain provides quick and trusted access by several actors, and facilitates the exchange of cross-border information. Authors solicit the use of IoT devices (laboratory and hospital devices) enabled to access directly the blockchain, without human intervention, so to further increase the level of trust in the platform. A token is issued to the account of people who have verified positive to antibodies characterized by an expiry date according to the expected age of antibodies. The system used biometric authentication, to enhance anonymity and privacy.

Finally, the DHP framework [62], proposes a private blockchain, where the Digital Health Passports (DHP) of citizens are registered and can be exploited for international tourism. The digital passport contains the result of a antibody tests, the timestamp specifying when the test is performed, the testing method. Unfortunately, the authors do not describe the cryptographic primitives used to link the DHP to the tested users. The blockchain is accessed by the Health Service Authorities of different countries, having full rights on the blockchain and by other authorized members which can only read data registered on it. The consensus algorithm is Proof of Authority.

## V. Blockchain for COVID-19 Beyond Contact Tracing and Vaccine Certification

In this section we briefly introduce some further blockchain-based applications for mitigating COVID-19 consequences.

Some interesting proposals [74], [75] combine machine learning and blockchain to define a federated or swarm learning approach. Reference [74] proposes a blockchain based federated learning framework to train and share a collaborative model. The objective of the proposed architecture is to train a global model by using locally trained models. Actual patients’ data are stored by the hospital and the blockchain helps to retrieve the trained models. Reference [75] exploits a private permissioned blockchain to coordinate the nodes of a Swarm Learning system. New nodes obtain the model, and perform local model training until defined conditions for synchronization are met. Then nodes exchange model parameters and a leader is dynamically elected, to perform the merge of the model parameters.

An interesting application to support social distancing is presented in [76]. The idea is to help health authorities to promote social distancing by controlling the number of individuals in specific areas. The blockchain is run by different government authorities. Citizens create a wallet where they receive “movement passes” or time-based tokens which can be spent and expire after a period of time. This way, the authorities can restrict the total number of tokens released in a certain period of the day for a certain area to limit the number of people in that area.

Finally, it is worth noticing that several blockchain-based applications for healthcare had already been proposed before COVID-19, and are currently very useful to face different aspects of the pandemics. [77] presents the main applications of blockchain in the healthcare area. One of the most important is the sharing of health records between different institutions, which is particularly complex, because of the presence of sensitive data. An example is MedRec [72], a permissioned blockchain for storing electronic healthcare records. Furthermore, blockchain can also assist the monitoring of patients through sensors and other IoT devices, by making the process more reliable. Finally, blockchain can be used to monitor the medical supply chain, in particular the distribution of vaccines.

## VI. Discussion and Challenges

In this section we first discuss some general issues of blockchain technology, then we present some considerations more strictly related to the use of this technology in the COVID-19 pandemic.

Even if blockchain is a promising technology, some issues are not yet completely resolved and deserve further research. The main one is related to the throughput of blockchain platforms, which may be too low for some applications and depends on the number of nodes participating to the protocol and number of transactions generated by them. A strictly related problem is that of transactions acceptance latency, dependent on the time needed to validate a block. To mitigate these problems, new consensus algorithms have been developed and are currently object of research. In particular, permissioned blockchains are characterized by a higher level of efficiency, since the number of participating nodes may be controlled and more efficient consensus algorithms may be adopted.

Another challenge is related to the trade-off between data auditability and privacy. All data published on the blockchain are public, so particular attention to sensitive data should be paid to fulfill privacy laws and regulations like the General Data Protection Regulation (GDPR). As shown in the previous sections, promising cryptographic techniques, like zero-knowledge proofs, can be exploited to retain the advantages of a blockchain, while ensuring the privacy of sensitive data.

As far as concerns the use of blockchains-based contact tracing solutions, some solutions presented in Sect. III present serious privacy threats. An example is that of the already discussed Paparazzi attack, which can be simply delivered exploiting silent tracing devices. If the attacker uses active devices (behaving as regular smartphones) more complex massive surveillance attacks can be performed. Therefore, the use of a blockchain is not, in itself, a panacea for contact tracing solutions. However, the use of a blockchain as a bulletin board where the pseudonyms of infected users are published, makes the entire process transparent and reliable and avoids attacks based on the collusion between the attacker and a centralized server. Using advanced cryptographic techniques, like Diffie Hellman or zero-knowledge proofs, combined with blockchain may guarantee stronger resistance to attacks and, at the same time, transparency.

Several location-based solutions exploit the Proof-of-Location mechanism which certifies the presence of a user at a location, at a certain time, where the witnesses are WIFI access points or other devices. However, a rewarding mechanism should be used to make this solution really feasible, since it is unrealistic that these devices would voluntary accept to use part of their resources, such as bandwidth, to implement PoL mechanisms. Furthermore, incentive strategies would help mitigating DoS attacks on such devices.

As for immunity certificates, even if they may favour the return to normal life for many citizens, [78], [79] observe that they may also raise several practical and legal challenges, because they give the privilege of working and participating to other social-related activities only to a the subset of certificated citizens. Close attention has to be paid also to the management of vaccine certifications, as they create disparities in the population. It is likely the certifications will be administered by government offices, and this may give rise to corrupt practices and bias towards a subset of citizens. Furthermore, dedicated legal regulations and protection are not yet available, so citizens cannot rely on legal certainty as a guarantee of their rights. The success of immunity and vaccine certifications will be largely dependent on the trust in the public authority, which, in many countries, can not be taken for granted. The use of blockchain technology, which provides trust in a trustless environment by design, can contribute to a wider popular acceptance of the use of these certifications.

## VII. Related Works

Even if the COVID-19 outbreak dates back just a year ago, the interest for technological solutions supporting the management of the pandemic has been very high. Some review articles [7], [12]–[16] have already presented several applications of blockchains for COVID-19.

Authors of [7] present an interesting statistical analysis of the main use cases of blockchain technology to mitigate COVID-19 challenges. The study is based on a search of scientific publications in the main bibliographic databases, looking for search terms related to the target technology. Nineteen eligible proposals are detected. Authors show that the most prominent use cases are contact tracing and immune/vaccine passports. Several interesting statistics are reported in this article, e.g. most applications use smart contracts on the Ethereum platform, and smart contracts are mainly developed in Solidity, and the second most used platform is Hyperledger. A part from the interesting statistics, this article neither describes the proposals in-depth nor shows any technical details. All the contact tracing and immunity passports proposals referred in [7] are investigated in depth in our survey.

The survey [12] reports a wide analysis of the main potential use cases pertinent to COVID-19. The work presents an high level description of the use cases, neither delving deep into the technological details nor presenting the technological challenges that these use cases present.

Reference [13] first introduces the general context of the COVID-19 outbreak, the main impacts of the pandemics on the global economy, and the clinical tests for COVID-19 detection. The last part of this article is devoted to the emerging technologies which may bring benefit to the management of the pandemics, i.e. IoT, drone technology, robots and autonomous vehicles, wearables, and blockchain. As such, only a small section of this article is dedicated to blockchain.

Also the survey [14] is an high level roundup of the main applications of blockchain technology for the COVID-19 pandemic, like disease control, traceability, supply chain of medical parts, and healthcare management. The technological side of these solutions is not investigated in this article.

References [15], [16] present comprehensive surveys of contact tracing applications for COVID-19 with particular focus on their privacy and security implications. However, [15] presents a single reference to a blockchain-based contact tracing application, i.e. PRONTO-C2, while [16] evaluates the current solutions on the basis of five parameters, i.e. centralization, proximity/GPS, privacy, adversarial model, and scalability, but this article does not consider the blockchain-based solutions.

## VIII. Conclusion

This article has presented an in-depth analysis of the recent blockchain-based solutions for COVID-19 contact tracing and for the management of immune/vaccine certifications. Contact tracing approaches have been classified according to the communication infrastructure they exploit: proximity based solution use mainly BLE, location-based solution may rely on GPS or Wifi, and some proposals also leverage the cellular network. We have shown how some proposals present serious security and privacy concerns. These issues can be overcome by using more advanced cryptographic techniques, like Diffie Hellman or zero-knowledge protocols. This article has also described blockchain-based solutions for immune/vaccine certifications, showing that a proper integration of self-sovereign identity systems with blockchain technology might enable to define privacy-aware and secure solutions.
